# Supplementary figures and images for: Local drivers of Rift Valley fever outbreaks in Mauritania: A one health approach combining ecological, vector, host and livestock movement data
Source: PLoS Negl Trop Dis. 2025 Sep 30;19(9):e0013553. doi: 10.1371/journal.pntd.0013553 (PMC12510638; doi:10.1371/journal.pntd.0013553)

## S1 Appendix

### Outbreak potential method workflow

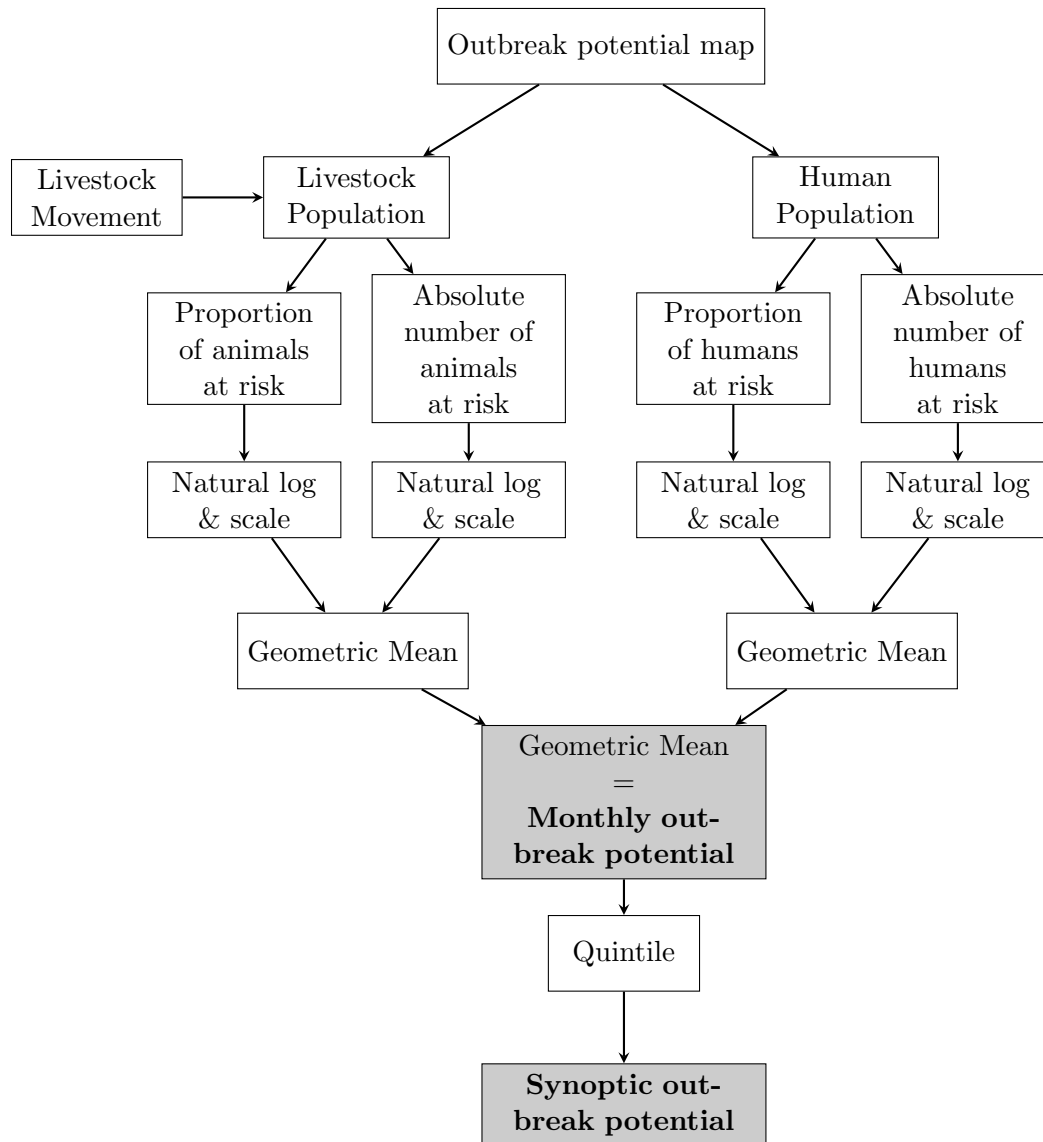

Supplement: S1 Appendix — (PDF) [file pntd.0013553.s001.pdf]
