# Supplementary material for: Local drivers of Rift Valley fever outbreaks in Mauritania: A one health approach combining ecological, vector, host and livestock movement data
Source: PLoS Negl Trop Dis. 2025 Sep 30;19(9):e0013553. doi: 10.1371/journal.pntd.0013553 (PMC12510638; doi:10.1371/journal.pntd.0013553)
Supplement: Attachment — Submitted filename: Response_reviewers_RVF_MRT.pdf [file pntd.0013553.s003.pdf]

Answer to reviewers' comments

Dear editor and reviewers,

Thank you for the time taken to review our manuscript, PNTD-D-25-00400, 'Local drivers of Rift Valley fever outbreaks and investigations of spillover to humans and animals in Mauritania: A One Health approach.'

Please find below and in the revised manuscript the answers to the reviewers' comments, which we have considered carefully.

Firstly, one major comment from reviewers pertained to the term 'spillover' used throughout the manuscript. The term “spillover” was inspired by the work of Hardcastle et al., 2020 (we quote):

“Given the seasonality of environmental suitability for RVF, we stratify risk at a monthly level, estimating environmental suitability from corresponding monthly climate data.

We combine suitability analyses with human and livestock population data to estimate the “spillover potential”. Combined with local considerations, these resources can guide where and when to focus RVF preparedness efforts.”

According to reviewers, the term “spillover” was misleading and confusing; thus, we decided to use two terms in the two models we produced:

- “ecological suitability for RVFV infection” - described by the ecological niche model we deploy at 1km resolution using all positive and negative occurrences of RVF in animals, humans and mosquitoes between 2019 and 2023 and climate and environment data in Mauritania

- “outbreak suitability for RVF emergence resulting in an epizootic/epidemic” - described by combining the ecological niche model with human, animal population data and livestock movements in Mauritania at 1km resolution.

Consequently, we changed the title and now it's: “Local drivers of Rift Valley fever outbreaks in Mauritania: A One Health approach combining ecological, vector, host and animal movement data” to underline better the multiple-source data used, including data on the animal movements, which was not done in previous works and the high resolution of the model outputs (local scale, 1km resolution).

The remaining answers to the reviewers are as follows.

Kindly yours,

Elena Arsevska, corresponding author

Montpellier, 18/07/2025

The manuscript by Barry et al. describes a model for investigating ecological drivers and spillover of the Rift Valley fever virus (RVFV) in Mauritania. The authors utilise recent data from samples collected between 2019 and 2023 in Mauritania in comparison to previous publications. With the aid of the established model, the authors determine that the precipitation of the current and the month before, as well as the average daily temperature of the current month, are the main drivers for RVF outbreaks in Mauritania. They could demonstrate that the wet season (August, September, and October) is the ecologically most favourable time for RVF occurrence and that spillover potential is highest during these months. According to the model, RVF occurrence begins in the southeastern region of Mauritania at the start of the wet season. Subsequently, it spreads to the southern area throughout the wet season.

In general, epidemiological models are highly valuable for better understanding the drivers of RVF outbreaks and spillover events. The topic is highly relevant for public health interventions, informed surveillance strategies, and effective intervention strategies. However, I am not convinced about the novelty of this study, nor the presentation of the data, for the reasons stated below.

Major comments:

The novelty of the manuscript does not become fully clear to me. A previous publication, referenced in the manuscript, demonstrates that RVFV transmission occurs during September and October in Mauritania, using a similar modelling approach (DOI: 10.3390/ijerph110100903). In addition, several models for RVFV occurrence prediction have been published (e.g., Caminade, 2014. DOI: 10.1073/pnas.0806490106, Anyamba, 2008, El-Nno Horn of Africa: DOI: 10.4269/ajtmh.2010.09-0289, Chemison, 22024: el-Nino Horn of Africa DOI: 10.1038/s41598-024-53774-x). Unfortunately, the manuscript does not address the similarities and differences with other models.

Thank you for the helpful remarks that we have now taken into account in the Introduction, which underscore the novelty and added value of the study.

Our study identifies the spatio-temporal risk of Rift Valley fever (RVF) ecological and outbreak potential based on two contributions:

i) the spatial-temporal distribution of favourable biotopes for the survival of primary mosquito vectors responsible for initiating RVF transmission (environmental suitability), and

ii) the spatio-temporal distribution of animal and human population, including animal movements coupled with the environmental suitability that leads to the occurrence of RVF outbreaks (outbreak suitability).

The agro-pastoral zones identified as environmentally suitable (see Figure 3 in the manuscript) are particularly notable for their temporary ponds, which provide optimal breeding conditions for primary RVF vectors such as Aedes vexans, with other vectors at the onset of the rainy season (usually August). Additionally, the zones identified as suitable for RVF outbreak occurrence facilitate better planning of active surveillance among health authorities.

Previous research by Caminade et al. (2014) characterised the rainfall and vegetation conditions (anomalies preceding) RVF outbreaks (in 1998, 2003, 2010 and 2012), finding that outbreaks in Mauritania usually occur in September and October, following a pattern of dry weather followed by heavy rainfall. Our study builds on this work by examining both the time and location of RVF outbreaks from a larger corpus of multiple source datasets, obtained through a collaboration between the public and animal health sectors at a high-resolution village level, utilising monthly data from 2019 to 2023. We analysed five consecutive years of surveillance data covering humans, animals, and mosquitoes, as well as animal movements, during both outbreak and non-outbreak periods. This comprehensive approach enabled us to create higher resolution risk maps that identify

areas of higher and lower risk of RVF ecological suitability and outbreak potential throughout Mauritania, per month and year and as long-term monthly estimates of risk to allow for better planning of active surveillance and awareness campaigns across public and animal health sectors."

Furthermore, our model outputs are fully exploitable for guided decision-making and surveillance planning (first and remaining co-authors of the work are in charge of surveillance in animal and human health sectors), as they are provided at a 1 km resolution and in a GeoTIFF file format, which can allow to place, for example, sentinel surveillance in time and space. In addition, Caminade's work did not take into account animal movements and their role in transmission, whereas we did. In conclusion, both works are complementary; Caminade provides precision on the dynamics preceding the outbreaks, while we offer spatiotemporal and ecological mapping of suitable ecological and outbreak zones.

Secondly, the work of Anyamba (2008) examines the impact of El Niño on the Horn of Africa (East Africa) and RVF outbreaks. Mauritania is more influenced by the Atlantic Ocean's sea surface temperature (SST) (Lafaye et al., 2013), which affects the dry and rainy seasons in Mauritania. Similarly, the modelling framework adopted by Chemison (2024) clearly states that El Niño Southern Oscillation (ENSO) and the Dipole Mode Index (DMI) that the authors explored correctly reproduce major RVF epidemics in Somalia, Kenya, Zambia and to a lesser extent, in Mauritania and Senegal - mainly because of the less critical effect of these oscillations/indexes for West Africa.

Third, in line 82, the authors mention that "…if the sampling or reception date was not given, the analysis date was used." It is unclear how large the subset of samples with unknown sampling dates is and when the analyses were conducted. If this subset is substantial and the analyses were performed, for example, during August, this could strongly bias the model.

We analysed data originating from a total of 906 sampling sites (621 negative, 285 positive). For negative samples, all sampling dates were given except for two ruminant sentinel sample sites in 2021. For positive samples, 38 samples (38 human cases in 2022) lacked a sampling date or reception date; therefore, the analysis date was used for them, assuming that the analysis date corresponds to the date of reception and sampling (as per the protocols for RVF human case management in Mauritania).
We provide precision on the dates and samples in the manuscript - see Section 32.1.1, Disease Data in the Materials and Methods.

Significant parts of the discussion can be transferred to the results section (e.g. from lines 302 and 312). Please explain in more detail the characteristics of the areas predicted to have a higher risk of spillover, addressing the following question: Is there a higher risk of spillover in rural or urban areas? How does the risk for spillover differ in areas with high pastoralism/livestock vs. low? How significant is the role of rivers and/or lakes in the risk of spillover? An addition to the manuscript would be to analyse these areas in more detail and compare them to one another. Several of these points are discussed, but could be scientifically substantiated.

The high risk of RVFV infection is primarily associated with rural areas, where extensive pastures and temporary marshes or ponds provide ideal breeding sites for mosquito vectors. The risk is particularly elevated within pastoralist systems, as herders often move with livestock that may be in the incubation period of RVF. Infected animals may be slaughtered and consumed with insufficient cooking, increasing the risk of zoonotic transmission.

In contrast, the risk is considered lower in urban areas, where people live in closer proximity and engage in small-scale family farming or peri-urban dairy farming. In peri-urban areas, livestock owners often apply insecticides to reduce insect populations, thereby limiting the contact between vectors and hosts.

Temporary ponds play a significant role at the start of the rainy season, though the duration of rainfall limits their influence. These habitats are highly suitable for primary RVF vectors such as Aedes vexans and Mansonia species. Additionally, secondary vectors such as Culex and Anopheles mosquitoes may be present, further contributing to transmission.

Conversely, rivers and their tributaries pose a moderate but persistent risk, which increases during the rainy season as flooding expands suitable breeding grounds. These areas are also typically used for agriculture, including the cultivation of rice and vegetables, which creates additional points of contact between humans, vectors, and animals. As effective biotopes for mosquito vectors, such environments can facilitate transmission of the RVF virus between animals and humans, maintaining the potential for spillover events. It has been demonstrated that precipitation and humidity are positively correlated with mosquito density, including primary vectors (Aedes and Mansonia) and secondary vectors collected in irrigation RVF areas, as observed in Kenya (Sang et al., 2016). That profile is the same in Mauritania since the first RVF outbreak occurred in 1987 (Saluzzo et al.,1989; Jouan et al., 1990). Continuous surveillance, particularly through sentinel animals for early warning, is crucial for monitoring viral circulation and mitigating the risk of outbreaks (see Figure 4 in the manuscript). An illustrative example is the limited RVF outbreak that occurred in 2018 on a Montbéliarde cattle farm in Rosso, Mauritania. On this farm, Montbéliarde cattle imported from France were severely affected by RVF, whereas local breeds exhibited only mild clinical signs of infection (Stoek et al., 2022).

Please discuss in more detail the role of mosquitoes in spillover and outbreaks. The disease data show only a very few RT-qPCR RVFV-positive mosquitoes, and these were only detected in October, which is a discrepancy from the predictions of the spillover model, where the potential for spillover was higher from August.

Indeed, in the dataset, we have very few RT-qPCR RVFV-positive mosquitoes.

Indeed, the mosquito data for the period before the outbreak potential were only available for August 2023 (all of them being negative). The same applies to the sentinel sheep and goats, which were also negative. We don't have the data available from August for the other years because no samples were collected (for mosquitoes).

From 2019 to 2023, we intervened late in sampling mosquitoes that are vectors of RVF. This is due to government mosquito control campaigns aimed at protecting public health, which use broad-spectrum insecticide spraying based on deltamethrin, during the week following the first reported case of a human or animal RFV case. It was only in September 2022 that our entomology team was able to carry out mosquito collection missions in time before the government instituted vector control measures for public health protection. In 2023, financial resources were insufficient to carry out the sampling of RVF mosquito vectors because the REDISSE Project had ended (which funded some of the sampling of vectors).

However, according to the model's results, the highest risk of spillover/emergence was predicted for September and October, starting in July and August, and gradually diminishing in November and December, not in August, as the reviewer wrote.

During the Rift Valley Fever (RVF) outbreaks in 2020 and 2022, the first confirmed animal cases of Rift were reported before any human cases. During investigations into the outbreaks, people interviewed stated that there was a surge in disease-carrying mosquitoes around temporary and semi-permanent ponds, where camels, cattle, and small ruminants drank and grazed near water collections. This favoured contact between susceptible hosts and potential vectors (Aedes vexans, Mansonia, and Culex quinquefasciatus). We lack the financial resources to monitor the sampled mosquitoes on an annual basis. After the incubation period, the disease manifests in animals through abortions in pregnant females, deaths of young animals, and significant morbidity in adults. Given the close contact

between herders and/or livestock owners and their animals, humans can become infected in several ways: by handling aborted fetuses when assisting females during abortion, by consuming raw products from sick animals (milk and meat), and also through bites from mosquitoes with vector competence and/or other mechanical vectors.

Additional comments:

- All Figures: More detailed figure descriptions would be helpful. Additionally, the legend fonts and axes fonts could be increased.

This has been addressed. Please verify the new figure legends.

- The model lacks disease data from the North of Mauritania. Please explain why and how this might influence the model.

The data from the northern part of the country (Chinguetti and Atar moughatas, which are part of Adrar wilaya) was used for analysis. Out of 48, there were 21 RVF-positive occurrences (in both humans, camels, and small ruminants) throughout the entire study period. These moughataa's involve both forms of farming and livestock breeding: transhumance and nomadism, directed towards the Southeast and the Valley of the Senegal River. Let us recall that during the rainy season (August to November), animals that are in transhumance and nomadism flee the Southeast and the Senegal River Valley areas to avoid arthropod bites (mosquitoes and flies). Since the North has an average annual rainfall between 50 and 100 mm in a normal year, this creates a milder climate with less vector proliferation due to the absence of favourable biotopes for the development of the various vector stages, except for the 2010 outbreak in Adrar (El Mamy et al., 2011).

The very north of Mauritania, Tiris Zemmour, has three departments: F'derick, Zouerat, and Birmoghrein. Dromedaries transported from the southeast of Mauritania to Tiris Zemmour for slaughter during the RVF outbreak in 2022 were found to be RT-qPCR positive for the RVF virus before slaughter. This highlights the role of animal mobility — facilitated by vehicles or other means of transport — in potentially introducing animals in the viremic and/or incubation phase into an RVF-free area.

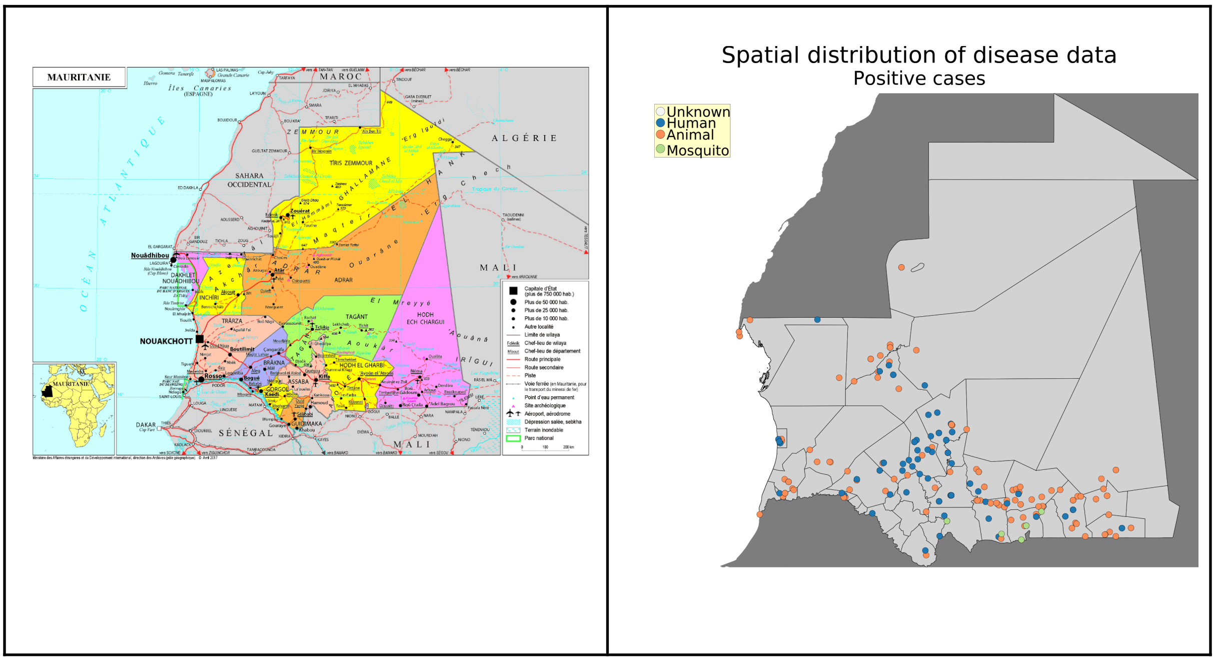

- Line 237: "In this study, we identified the ecological factors associated with RVF outbreaks in Mauritania…": please formulate less strongly, as not all ecological factors were considered in this model (e.g. wind).

We agree with the reviewers. The phrase has been modified.

- Line 294: "…since small ruminants positive for IgM were detected…": better write "…since small ruminants with anti-RVFV IgM antibodies…" or "…seropositive for RVFV…".

We agree with the reviewers. The phrase has been modified.

Editor comments

The reviewers have raised several substantive concerns that must be addressed before the manuscript can be considered for publication. The most significant issue raised across the reviews relates to the novelty and added value of your proposed model. While the work is well-structured and technically sound, the reviewers noted that it is essential to clarify and justify how your model meaningfully advances the current state of the art, whether in terms of methodological innovation, predictive accuracy, spatial or temporal resolution, or practical applicability in public health settings.

Regarding the novelty of the study, please refer to the revised introduction of the manuscript, which highlights existing works and the added value of our contribution.

In addition, the first author of this work noted the following added value and practical implications in the field, which we have also incorporated into the revised conclusions:

• Raise awareness among relevant partners before the abnormal rainfall events and during the rainy season and months and areas identified at risk of infection and outbreaks to take measures to protect at-risk personnel (breeders, owners, butchers, veterinary staff and brokers, etc.);

• Raise awareness among decision-makers about the negative socioeconomic consequences (human deaths, loss of livestock, and loss of livestock renewal due to mass mortality of young animals associated with abortions leading to stillbirths and an embargo on the sale of meat and live animals, etc.)

• Crisis management (implementation of an emergency response plan and conducting simulation exercises on hemorrhagic fevers, etc.) in areas identified as high risk

• Develop guides and manuals (provision of guidelines to follow in cases of suspected RVF and other fevers at veterinary and human health centres, with a clear definition of RVF cases)

• Popularise the manuals (disseminate a manual to professionals and others)

According to PLOS policies, all data must be publicly available; however, one of the reviewers noted that the data used is not available.

The data, aggregated per wilaya, can be made available upon personal request from the first author. We added this statement to the manuscript in the data and code availability section.

Journal Requirements:

1) Please ensure that the CRediT author contributions listed for every co-author are completed accurately and in full.
At this stage, the following Authors/Authors require contributions: Yahya Barry, Markus Metz, Lina Krisztian, Julia Haas, Victoria-Leandra Brunn, Abdellahi Diambar Beyit, Ahmed El Bara, Ahmed Bezeid El Mamy Beyat, Habiboulah Habiboulah, Markus Neteler, and Catherine Cêtre-Sossah. Please ensure that the full contributions of each author are acknowledged in the "Add/Edit/Remove Authors" section of our submission form.

The list of CRediT author contributions may be found here:
https://journals.plos.org/plosntds/s/authorship#loc-author-contributions

Thank you for the clarification and guidance. The contributions were adjusted according to the PLOS ONE CRediT author contributions - in the Overleaf document and accordingly in the submission portal, as can also be seen below:

Conceptualisation: EA, MN, MM, LK, JH, VB. Methodology: EA, MN, MM, LK, JH, VB. Data Curation: BY, ADB, AEB. Formal Analysis: MM, LK, JH, VB. Writing – Original Draft Preparation: EA, LK, JH, VB, MM, MN, CSS, BY. Writing – Review & Editing: BY, CSS, ADB, AEB, ABEMB, HH.

2) We ask that a manuscript source file is provided at Revision. Please upload your manuscript file in one of the following formats: .doc, .docx, .rtf, or .tex. If you are providing a .tex file, please upload it under the item type "LaTeX Source File" and leave your .pdf version as the item type "Manuscript".

Agree, we proceeded according to the suggestion during the submission portal.

3) Please provide an Author Summary. This should appear in your manuscript between the Abstract (if applicable) and the Introduction, and should be 150-200 words long. The aim should be to make your findings accessible to a broad audience, including both scientists and non-scientists. Sample summaries can be found on our website under Submission Guidelines:
https://journals.plos.org/plosntds/s/submission-guidelines#loc-parts-of-a-submission

4) We do not publish any copyright or trademark symbols that usually accompany proprietary names, eg ©, ®, or TM (e.g. next to drug or reagent names). Therefore, please remove all instances of trademark/copyright symbols throughout the text, including:
- ® on page: 3.

Done, thank you for the suggestion.

5) Please upload all main figures as separate Figure files in .tif or .eps format. For more information about how to convert and format your figure files please see our guidelines:
https://journals.plos.org/plosntds/s/figures

Done, thank you for the suggestion.

6) We noted that there are 4 figures included in the manuscript; however, there are 5 figures uploaded in the online submission form. Please check the figures labels and citations. Please also ensure that all Figure files have corresponding citations and legends within the manuscript. Currently, Figure 5 in your submission file inventory does not have an in-text citation.

We saw that it was probably the schema of the Spillover method from the appendix. We don't need this uploaded as an extra figure. All four figures for separate upload as .tif are also now available within Overleaf in the folder "figures/tif/".

7) We noted that there is a reference to " Appendix A" on page 7; however, there is no corresponding file uploaded to the submission. Please note that the supplementary files should be uploaded as separate files with the item type 'Supporting Information'.

We moved the appendix from the manuscript to a separate file, which can now be uploaded separately. It's called "appendix_A.tex"

8) Some material included in your submission may be copyrighted. According to PLOSu2019s copyright policy, authors who use figures or other material (e.g., graphics, clipart, maps) from another author or copyright holder must demonstrate or obtain permission to publish this material under the Creative Commons Attribution 4.0 International (CC BY 4.0) License used by PLOS journals. Please

closely review the details of PLOS's copyright requirements here: PLOS Licenses and Copyright. If you need to request permissions from a copyright holder, you may use PLOS's Copyright Content Permission form.
Please respond directly to this email and provide any known details concerning your material's license terms and permissions required for reuse, even if you have not yet obtained copyright permissions or are unsure of your material's copyright compatibility. Once you have responded and addressed all other outstanding technical requirements, you may resubmit your manuscript within Editorial Manager.
Potential Copyright Issues:
i) Figures 1, 3, and 4. Please (a) provide a direct link to the base layer of the map (i.e., the country or region border shape) and ensure this is also included in the figure legend; and (b) provide a link to the terms of use / license information for the base layer image or shapefile. We cannot publish proprietary or copyrighted maps (e.g. Google Maps, Mapquest) and the terms of use for your map base layer must be compatible with our CC BY 4.0 license.
Note: if you created the map in a software program like R or ArcGIS, please locate and indicate the source of the basemap shapefile onto which data has been plotted.
If your map was obtained from a copyrighted source, please amend the figure so that the base map used is from an openly available source. Alternatively, please provide explicit written permission from the copyright holder granting you the right to publish the material under our CC BY 4.0 license.

Base layer of maps in Figures 1,3,4: *Administrative areas (boundaries), country outlines, and administrative subdivisions for Mauritania were obtained from the GADM Vector dataset from https://gadm.org/download_country.html and licence https://gadm.org/license.html*

GADM license
The data are freely available for academic use and other non-commercial use. Redistribution or commercial use is not allowed without prior permission.

Using the data to create maps for the publishing of academic research articles is allowed. Thus, you can use the maps you created with GADM data in figures for articles published by PLOS, Springer Nature, Elsevier, MDPI, and other reputable publishers. You are allowed (but not required) to publish these articles (and the maps they contain) under an open license such as CC-BY, as is the case with PLOS journals and may be the case with other open-access articles. Data for the following countries is covered by a different license Austria: Creative Commons Attribution-ShareAlike 2.0 (source: Government of Austria)

9) Please amend your detailed Financial Disclosure statement. This is published with the article. It must therefore be completed in full sentences and contain the exact wording you wish to be published.

Done

1) Please clarify all sources of financial support for your study. List the grants, grant numbers, and organizations that funded your study, including funding received from your institution. Please note that suppliers of material support, including research materials, should be recognized in the Acknowledgements section rather than in the Financial Disclosure

Done

2) State the initials, alongside each funding source, of each author to receive each grant. For example:

"This work was supported by the National Institutes of Health (####### to AM; ###### to CJ) and the National Science Foundation (###### to AM)."

Done

3) State what role the funders took in the study. If the funders had no role in your study, please state: "The funders had no role in study design, data collection and analysis, decision to publish, or preparation of the manuscript."

Done

4) If any authors received a salary from any of your funders, please state which authors and which funders.

None received

10) Your current Financial Disclosure states, "The author(s) received no specific funding for this work.".
However, your funding information on the submission form indicates receiving a fund. Please ensure that the funders and grant numbers match between the Financial Disclosure field and the Funding Information tab in your submission form. Note that the funders must be provided in the same order in both places as well.

Done

11) The file inventory includes files for Figures 1a, 1b, 2a and 2b. We would recommend either combining these into single Figure 1.tiff and Figure 2.tiff files with separate internal panels, or renumbering them as individual figures, as we are not able to publish multiple components of a single figure as separate files.

Done. Combined to Fig1 and Fig2

Comments to the Authors:

Reviewer #1: The objectives of this study are simple and well stated. I had to admit that I lack more than basic competence in the type of modelling applied in this study (but so also do many potential readers), and I think some clarification on the sources of the data used would be helpful:

1. It appears that the humans and animals sampled were largely (exclusively?) from areas where RVFV infections had been formally recognised, which might make conclusions about other areas somewhat weak.

Active surveillance for RVF in Mauritania is carried out by installing and monitoring sentinel herds throughout the rainy season. Each herd is identified using ear tags bearing a unique number. The herd consists of between 15 and 20 animals under one year of age. Monitoring is conducted over three months (typically between August and October), with blood sampling performed on each animal every month. Sentinel herds and sampling sites are identified and monitored by a local veterinarian inspector. The sites are chosen based on each veterinary inspector's experience and knowledge of the RVF circulation (from previous outbreaks or results from project field studies) and areas that might be suitable biotopes for mosquito vectors (not experiencing RVF outbreaks in the past) together with a population of susceptible animals, such as sheep, goats, cattle, or camels. Additionally, the villages serving as sampling sites are also accessible by road or on foot.

During active surveillance, an effort is made to select locations from the same moughataa (administrative level 3) or villages that were previously monitored within this Moughataa or in their proximity, fulfilling the conditions regarding biotopes, animal presence, and field accessibility. Active

surveillance involves testing for IgM antibodies in small ruminants (typically sheep and goats), indicating the active circulation of the RVF virus.

Event-based or passive surveillance is ongoing through the network of informant farmers and the area's veterinary services to report any abnormal disease and symptoms (usually massive abortions and mortality) observed in animals. Informant farmers are the key link in the field system within the framework of the Mauritanian Network for Epidemiological Surveillance of Animal Diseases in Mauritania (REMEMA). The local population selects informant farmers to serve as relays or informants at the nearest veterinary station.

Mosquito surveillance is, however, mainly conducted depending on funding (usually from projects) and during the confirmation of RVF outbreaks.

From 2019 to 2023, we intervened late in sampling mosquitoes that are vectors of RVF. This is due to government mosquito control campaigns aimed at protecting public health, which use broad-spectrum insecticide spraying based on deltamethrin, during the week following the first reported case of a human or animal RFV case. It was only in September 2022 that our entomology team was able to carry out mosquito collection missions on time, before the government instituted vector control measures for public health protection. In 2023, financial resources were insufficient to carry out sampling of RVF mosquito vectors because the REDISSE Project had ended (which funded some of the sampling of vectors).

2. Human sampling appears to have been limited to hospitalised patients (again from areas known to be undergoing RVF outbreaks) - does this not bias the sample base for the analyses that were done? Could it be that RVFV is circulating in areas that were judged to be at very low risk (northern Mauritania)? Also, as the authors describe, RVF is often very mild in humans, and such patients would not likely visit a hospital.

Human sampling remains limited to consultations with health facilities or patients suspected of being infected with RVFV; their serum is sent to the INRSP (National Institute for Public Health Research) along with a complete anamnesis of this patient. Therefore, any patient suspected of having a hemorrhagic fever undergoes tests using the ELISA Test and RT-qPCR, particularly for RVF, CCHF, and Dengue.

3. 25% of the camels were RVFV positive by RT-PCR - this looks high considering the transient nature of viremia for this disease. Please clarify the criteria for sampling camels, and the specific samples (sera?) used for testing.

This percentage of positive dromedaries is due to obtaining fresh samples from the field immediately after blood sampling. This is because a large number of dromedaries are sampled early upon the appearance of a sick animal in the herd. This is why there may be animals sampled at the very beginning of viremia. The RT-QPCR used is a test recommended by the WHO (Reference Manual of the WHO).

4. Mosquitoes: First, were they tested individually or, as is more commonly done, as pools? Second, were these mosquitoes sampled only from areas with animal or human cases?
Expand the description of the sampling strategy to address the questions I pose above. Additionally, if necessary, include some items in the limitations section, which is generally quite comprehensive.

WRITE AN ANSWER: Les moustiques sont testés par un pool de 5 individus de la même espèce, du même site d’échantillonnage. les techniques d’extraction sont les mêmes techniques utilisées pour écraser les tissus animaux ou autre par le kit Qiagen en utilisant Thermotissulyser avec des billes ‘ une

à 2 billes par tubes contenant 5 moustiques femelles de même espèces. Des essais de pools sur les moustiques mâles ont été effectués mais leurs résultats étaient négatifs.

Reviewer #2: Pooling livestock, human, and mosquito RVFV-positive data will not inform spillover risk. There needs to be a location-specific consideration that accounts for the expected lag between livestock and human cases. Spillover by definition, is the escape of the virus from one reservoir to another -- in this case, livestock to humans.

We acknowledge that the term 'spillover' was misleading in this work; we provide a new definition, as stated in the introduction to the answer to the reviewers and in the Materials and Methods section.

The first author of this work provides further explanation:

In 2022, active surveillance of the RVF virus was done with sentinel herds and the suspicion of RVF in dromedaries was confirmed by the RT-qPCR test, three (3) weeks before the proven human cases of RVF. As soon as the sentinel herds and the confirmation of the suspicion of RVF in dromedaries, the members of the One Health platform were convened either in person or online by videoconference to share the results of the recent circulation of the virus in animals to inform the human health and environment sector of the circulation of the RVF virus in animals to take response measures to minimize infections and deaths in humans. This is two weeks after there were human cases with a reduced number of deaths compared to previous outbreaks of RVF in Mauritania (1998, 2010, 2012, 2015, 2020).

Secondly, why did we group all the cases? The idea was to superimpose the positive results of Rift in humans and animals, in addition to the data on mosquitoes, to assess the significance of this data superposition in relation to the climatic data and establish a positive correlation, based on the level of risk in the area in question. As mentioned earlier, in 2022, the first index case was a dromedary, with the sentinel herd revealing the active circulation of RVF before the proven human case of Rift, which occurred two weeks prior. Therefore, we say that the spill-over could occur from close contact between humans and infected animals, either directly or indirectly, by using the milk or meat of sick animals or in the incubation phase during slaughter or abortion.

By pooling data from the two reservoirs, I don't see how conclusions on spillover can be made - did I miss something? Pools of mosquitoes of the same species from the same sampling site were used. One mosquito pool had 5 individuals of the same species.

Reviewer #3: The objectives are clearly articulated.
The study design could be improved (see attachment for my comments).
The population is clearly described and appropriate.
The sample size is sufficient.
No statistical analyses were included because they were deemed unnecessary.
There are no concerns about ethical or regulatory requirements.

Thank you very much

Results

Reviewer #1: Results are clear and make sense; you might indicate (if you agree) that the highest value predictor (rainfall) is actually a surrogate for mosquito populations.

We agree. This is since precipitation fills ponds depending on their intensity and frequency. Once the ponds are full of water, they remain breeding grounds with a microclimate favourable to the

emergence of the primary vectors of RVF (Aedes vexans). The relay of transmission of the RVF virus is then ensured by the population of Culex mosquitoes and other competent vectors, such as Mansonnia sp. and Anopheles. Since these ponds serve as watering and grazing areas for domestic ruminants and humans, they facilitate encounters between susceptible hosts and the mosquitoes that carry RVF.

For the large versions of the spatial maps (end of manuscript), the text legends are quite blurry - it would be great if the text in those images could be made more clear.
Figure 2: It would seem to be much more clear if the x-axis on both positive and negative graphs were the same to make comparisons easier.

Figures updated in the manuscript

Reviewer #2: (No Response)

Reviewer #3: The analysis matched the objectives. Nevertheless, a major concern lies within the given RT-qPCR and serological data.

First, the authors only mention a reference for the RT-qPCR data for the camels and for the mosquitoes. For the RT-qPCR for the human samples and for the ELISA data there is neither a reference given nor the original data shown in the manuscript.

→ Lines 64-66 of the Disease data section, the commercial kit that was used for genomic detection by RTqPCR for human samples is indicated: the RealStar® Rift Valley Fever Virus RT-PCR Kit 1.0 (Altona Diagnostics GmbH, Germany). No ELISA was performed on human samples. To clarify, in Lines 72-73 of the Disease data section, the RT-PCR method used to detect the RVF genome in animal samples is based on the paper by Bird et al. (2007) (REF 15).

Second, while human, camel, and mosquito samples were tested by RT-qPCR, cattle, sheep, and goats were tested by ELISA. Hence, provision of the assay results and a consistent testing method would be desirable. Confirmation of ELISA data by a neutralisation test would strengthen the manuscript, but it is not essential.

→ The authors agree with the reviewer. It would have been beneficial to test samples from all animal species (cattle, sheep, goats, and camels) using a unique method (i.e., the IgM ELISA method). Still, the only RVF IgM ELISA kit commercialised does not work in the camel species; this is the reason why camel samples have been tested through the RT-PCR method rather than IgM ELISA. The neutralisation test requires the use of routine cell culture, which is not the case in the two laboratories in Mauritania. Testing the sera for neutralisation can be done by sending the samples to an international reference laboratory that is capable of performing such tests. We do not choose this option as the specificity and sensitivity of the RVF IgM capture ELISA used are high (Wilson et al., 2013); this test is used in routine sentinel herds for RVF passive surveillance in the country. We are confident in the IgM data obtained, which corroborates with the PCR data *(Wilson et al; 2013 the DOI is not available but there is the title: Diagnostic Approaches for Rift Valley Fever)*

Human RVFV diagnosis was performed at INRSP, accounting for a first step of RNA extraction using the QI95 Aamp viral RNA kit (Qiagen, USA), followed by an RT-qPCR using the RealStar® Rift Valley Fever Virus RT-PCR Kit 1.0 (Altona Diagnostics 96 GmbH, Germany) in a Rotor-Gene Q system (Qiagen USA) following manufacturer's instructions. Standard RT-PCR assays are available for both mammalian and mosquito samples (Anonymous, 2008)

All Figures should be revised. More detailed figure descriptions would be helpful. Additionally, the legend fonts and axes fonts should be increased.

Figures legend + axes + title updated in manuscript. More detailed figure description added.

Editorial and Data Presentation Modifications

Abstract: “high-resolution spatiotemporal scale” –> temporal scale model?

We still prefer to keep the term spatio-temporal scale because the model outputs were indeed spatial and temporally varying.

"molecular biology" -> virus RNA detection

We agree the change has been made.

2.3 section on RVF spillover potential for humans and animals: in this section, there are issues with tense: you change from describing was/were to is/are (they should be was/were)

Reviewer #2: The separation of RVF infection data into spatial and temporal categories, and within each category, into positive and negative, is not helpful because reservoirs (i.e., livestock vs. human) are pooled.

Separation into positive and negative is essential to train the model (also explained in the methods)

There is no information available to the reader that explains how a lag between livestock and human cases could support the understanding of patterns of spillover.

For one location of infection, I can determine who was the first positive, whether it was an animal, human, or mosquito, and calculate the lag. We were examining the overall spillover, regardless of species, based on the available and most recent data. Explain the methods more clearly to clarify what we consider a spillover and its significance.

Reviewer #3: - Line 237: “In this study, we identified the ecological factors associated with RVF outbreaks in Mauritania…”: please formulate less strongly, as not all ecological factors were considered in this model (e.g. wind).

We agree. Now, this phrase has been reformulated into: “In this study, we explored a subset of ecological factors associated with RVF outbreaks in Mauritania, which allowed us to identify areas with environmental conditions that may support the occurrence of the disease. Additionally, by incorporating available data on population distribution and animal movement within Mauritania, we outlined areas with a potential risk for RVF outbreaks in humans and animals.” (LXX).

- Line 294: “…since small ruminants positive for IgM were detected…”: better write “…since small ruminants with anti-RVFV IgM antibodies…” or “…seropositive for RVFV…”.

We agree. Now, this phrase has been reformulated into: “These results are not unexpected, as small ruminants seropositive for RVFV were detected during sentinel surveillance in December 2019 and January 2020 in Nouadhibou. Additionally, Hodh El Gharbi is also represented in our dataset (author’s communication)”.

Summary and General Comments

Reviewer #1: This is a useful description of modeling to better describe risk factors for RVF in Mauritania. Overall, the methods used appear appropriate, but some clarification on the data sets used in the analyses would be valuable.

- *Negative: 6 unknown, 132 Mosquito, 326 Animal, 157 Human*
- *Positive: 0 unknown, 8 Mosquito, 161 Animal, 116 Human*

Reviewer #2: The authors are showing RVFV transmission risk in general (to livestock and humans), and there is not enough evidence (at least in the current way it is presented and explained) for me to see a fine-scaled description of spillover risk.

During the introduction of RVF in 1987, following the filling of the Diama dam, the observation was that there was an intense proliferation of mosquitoes following the flooding of arid areas, thus creating favourable conditions for contact between susceptible domestic ruminants with vector mosquitoes and humans. This situation has remained almost identical to all outbreaks reported in Mauritania, either after a year or years of drought followed by rainfall whose average is above normal in one or neighboring regions (Diallo et al., 2005; EL Mamy et al. 2010, Sow et al., 2014, and Barry et al., 2022,) on the one hand. Compared to the sensitivity of domestic ruminants, would these be due to the loss of immunity during inter-epidemic periods of RVF and according to the virulence of the RVF strain? Furthermore, the life expectancy of a small ruminant is, on average, 4 to 5 years (RISSMANN et al., 2016) or even 7 years at most. Furthermore, there is no chronic carrier state for the RVF virus (World Organisation for Animal Health, 2019).

Reviewer #3: In general, epidemiological models are highly valuable to better understand the drivers for RVF outbreaks and spillover events. The topic is highly relevant for public health interventions and informed surveillance strategies as well as intervention strategies. However, I am not convinced about the novelty of this study as well as about the presentation of the data.
The manuscript describes a model for investigations of ecological drivers and spillover of Rift Valley fever virus (RVFV) in Mauritania. The authors use, compared to previous publications, recent data from samples collected between 2019 and 2023 in Mauritania. It seems like the authors main novelty is the use of recent data. The model which was established newly, did not come to any different conclusions than other models used before. The main results, that the wet season is a main driver of RVF occurrence and spillover in Mauritania, were seen before with other models.

The importance of our study lies in characterising areas at very high, high, medium, and negligible risk, based on an ecological suite of covariates that can result from either heavy rainfall or extensive flooding. Furthermore, the vegetation index remains too limited, given that there are large pasture areas without temporary or semi-permanent ponds. In this context, neither animals nor humans can stay there for long. This is why our model provides more information on biotopes and/or microclimate zones favourable to the development of competent vectors. Moreover, these ecozones are favourable locations for encounters between vectors and susceptible animal hosts (dromedaries, sheep, goats, and cattle). This is in line with existing livestock practices in Mauritania (transhumance and nomadism). Furthermore, there have been years of rainfall, for example, in 2009. Still, the quantities of water did not accumulate in areas with temporary pools that produced Aedes vexans and other mosquitoes, and there were no outbreaks of RVF. Around the pools that serve as breeding grounds for the vectors (Aedes vexans and Culex poicilipes) of Rift Valley fever (RVF), a variable environment is created where mosquitoes can thrive and contribute to its spread and transmission in the Ferlo (NDione et al., 2009)

Additionally, the raw data for the virological assays are missing. These data should be made publicly available.

Data can be made available upon request from the first author.

To improve the manuscript, e.g. the influence of human-livestock interaction (consumption of meat, unpasteurized milk, husbandry) could be further explored. In addition, the role of mosquitoes should be assessed in more detail. Besides this influence of rural vs urban areas, proximity to lakes or rivers could be included in the model and assessed in more detail (for further explanations, please see my attached document).

In the discussion, we emphasise the importance of peri-urban centres and livestock production areas. Please check if it's clearer now.
