# Supplementary material for: Local drivers of Rift Valley fever outbreaks in Mauritania: A one health approach combining ecological, vector, host and livestock movement data
Source: PLoS Negl Trop Dis. 2025 Sep 30;19(9):e0013553. doi: 10.1371/journal.pntd.0013553 (PMC12510638; doi:10.1371/journal.pntd.0013553)
Supplement: Attachment — Submitted filename: Barry et al_PLoS neg trop dis 2025_Review.docx [file pntd.0013553.s002.docx]

The manuscript by Barry et al. describes a model for investigations of ecological drivers and spillover of Rift Valley fever virus (RVFV) in Mauritania. The authors use, compared to previous publications, recent data from samples collected between 2019 and 2023 in Mauritania. With the aid of the established model, the authors determine that the precipitation of the current and the month before as well as the average daily temperature of the current month as the main drivers for RVF outbreaks in Mauritania. They could show that the wet season (August, September, and October) is the ecologically most favourable time for RVF occurrence and that spillover potential was the highest during these months. According to the model, RVF occurrence starts in the southeastern region of Mauritania in the beginning of the wet season and further distributes over the southern area in the course of the wet season.

In general, epidemiological models are highly valuable to better understand the drivers for RVF outbreaks and spillover events. The topic is highly relevant for public health interventions and informed surveillance strategies as well as intervention strategies. However, I am not convinced about the novelty of this study as well as about the presentation of the data for reasons stated below.

Major comments:

1. The novelty of the manuscript does not become fully clear to me. A previous publication, which was mentioned in the manuscript as reference, shows that RVFV transmission occurs during the months of September and October in Mauritania, similarly with a modelling approach (DOI: 10.3390/ijerph110100903). In addition to this, several models for RVFV occurrence prediction have been published (e.g. DOI: 10.1073/pnas.0806490106, DOI: 10.4269/ajtmh.2010.09-0289, DOI: 10.1038/s41598-024-53774-x). Unfortunately, similarities and differences to other models were not addressed in the manuscript.
2. A major concern lies within the given RT-qPCR and serological data. First, the authors only mention a reference for the RT-qPCR data for the camels and for the mosquitoes. For the RT-qPCR for the human samples and for the ELISA data there is neither a reference given nor the original data shown in the manuscript. Second, while human, camel, and mosquito samples were tested by RT-qPCR, cattle, sheep, and goats were tested by ELISA. Hence, provision of the assay results and a consisting testing method would be desirable. Confirmation of ELISA data by a neutralization test would make the manuscript stronger, but is not essential.

Third, in line 82 they authors mention that “…if the sampling or reception date was not given, the analysis date was used.”. It is not clear how big the subset of samples with unknown sampling date is and when the analyses were conducted. If this subset is substantial and the analyses were conducted e.g. during August, this could strongly bias the model.

3. Significant parts of discussion part can be transferred to the results section (e.g. from lines 302, 312). Please explain in more detail the characteristics of the areas that were predicted to have higher risks of spillover with regard to the following questions: Is there a higher risk for spillover in rural or urban areas? How does the risk for spillover differs in areas with high pastoralism/livestock vs. low? How big is the role of rivers and/or lakes to the risk of spillover? An addition to the manuscript would be to analyse these areas in more detail and compare them to one another. Several of these points are discussed, but could be scientifically substantiated.
4. Please discuss in more detail the role of mosquitoes for spillover and outbreaks. The disease data show only very few RT-qPCR RVFV positive mosquitoes and only in October, which seems to be a discrepancy to the predictions of the spillover model, in which potential for spillover was higher from August.

Additional comments:

- All Figures: More detailed figure descriptions would be helpful. Additionally, the legend fonts and axes fonts could be increased.
- The model lacks disease data from the North of Mauritania. Please explain why and how this might influence the model.
- Line 237: “In this study, we identified the ecological factors associated with RVF outbreaks in Mauritania…”: please formulate less strong, as not all ecological factors were considered in this model (e.g. wind).
- Line 294: “…since small ruminants positive for IgM were detected…”: better write “…since small ruminants with anti-RVFV IgM antibodies…” or “…seropositive for RVFV…”.
